# Reactive Granulomatous Dermatitis in a Child with Acute Lymphoblastic Leukemia

**DOI:** 10.1155/2023/3428162

**Published:** 2023-10-12

**Authors:** Hannah C. Tolson, Mariana McCune, Miranda Yousif, David DiCaudo, Elizabeth Dupuy

**Affiliations:** ^1^University of Arizona College of Medicine Phoenix, Phoenix, Arizona, USA; ^2^Mayo Clinic Scottsdale, Phoenix, USA; ^3^Phoenix Children's Hospital, Phoenix, USA

## Abstract

Reactive granulomatous dermatitis (RGD) is an umbrella term to describe a reaction pattern characterized by skin-colored to erythematous papules, plaques, and nodules although other morphologies have been described. RGD has rarely been reported in children, and in this report, we present the case of a 3-year-old girl with acute lymphoblastic leukemia (ALL) who presented with firm, tender nodules, and ulcerated plaques on her extremities. Histopathologic examination showed foci of dense granulomatous inflammatory infiltrates composed of histiocytes, neutrophils, and multinucleate giant cells. The constellation of clinical symptoms, negative infectious workup, and histopathology support the diagnosis of RGD.

## 1. Introduction

RGD is a rare inflammatory dermatosis that is typically associated with underlying diseases [1]. Clinical symptoms usually involve multiple discrete, 2–10 mm plaques, or papules with central umbilication or necrosis; the distribution favors the extensor surfaces of the extremities, but it can present on the trunk and face [2, 3]. RGD occurs most frequently in the setting of inflammatory conditions such as lupus, rheumatoid arthritis, or systemic sclerosis; however, more recent cases describe its cooccurrence with hematologic malignancies [1, 2, 4]. There is a paucity of literature regarding RGD in the pediatric population. In this report, we present the first case of RGD in a pediatric patient with ALL and review other cases of pediatric RGD.

## 2. Case Presentation

**Figure 1:**
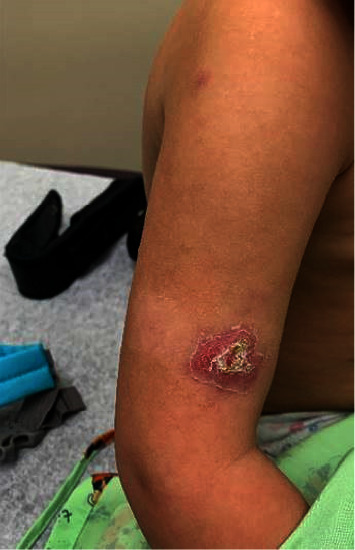
Clinical image demonstrating erythematous papule on the right posterior shoulder and larger erythematous plaque with white-yellow scale and crust.

A 3-year-old female with a history of ALL presented to dermatology with 2-month history of papules on her upper and lower extremities that would intermittently ulcerate and drain purulent fluid. At the time of presentation, the patient was several months into maintenance chemotherapy for ALL. She had completed induction and consolidation chemotherapy with IV vincristine, IV PEG-asparaginase, intrathecal cytarabine, intrathecal methotrexate, and oral dexamethasone a year prior. She completed interim maintenance and delayed intensification with IV vincristine, oral 6-mercaptopurine, and intrathecal methotrexate six months prior. She was on maintenance chemotherapy with IV vincristine, oral dexamethasone, and intrathecal methotrexate given in cycles every three months along with weekly oral methotrexate and daily oral 6-mercaptopurine. She started her most recent cycle of maintenance chemotherapy including oral dexamethasone two weeks prior to the eruption of skin lesions. The patient was on dexamethasone for 5 days before discontinuing. Additional history revealed that the patient contracted parainfluenza virus prior to the onset of skin lesions. She had been treated empirically with triamcinolone ointment, mupirocin ointment, and neosporin without relief. On examination, she had several scattered small erythematous papules and nodules on the extensor surfaces of her extremities, including some with yellow-white scale and crust, as shown in Figure 1. In addition, she had few smaller subcutaneous pink-to-violaceous nodules on the lateral thigh and calf.

A skin biopsy performed by an outpatient general dermatologist prior to referral to our clinic showed collections of neutrophils surrounded by histiocytes, some of them multinucleate and arranged in nodules. After the initial skin biopsy, the patient's disease progressed and she developed worsening redness, swelling, and pain of her lesions for which she was treated with oral clindamycin due to concern for cellulitis.

**Figure 2:**
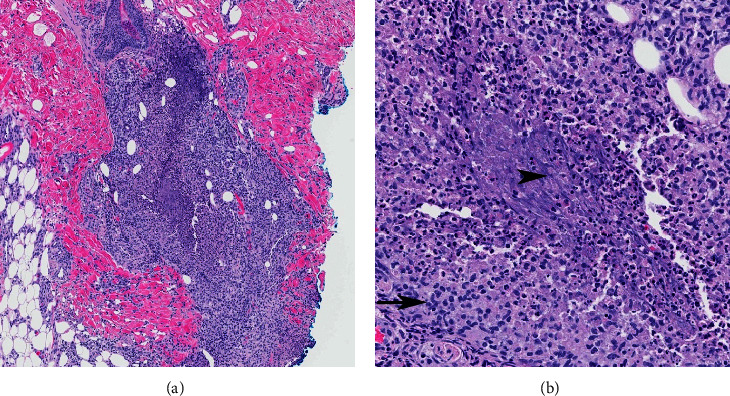
Granulomatous dermal inflammation with central basophilic necrosis and neutrophils (arrowhead) and a peripheral zone of palisading histiocytes (arrow). Hematoxylin-eosin; original magnification at x100 (a) and x400 (b).

Skin biopsy was repeated which revealed focally dense mixed granulomatous inflammatory infiltrates composed of histiocytes, neutrophils, and focal multinucleate giant cells in the subcutis and dermis. Neutrophils and karyorrhectic debris were associated with the necrotic tissue in the centers of palisading granulomas. The leading histopathologic considerations included palisaded neutrophilic and granulomatous dermatitis or infection, as shown in Figure 2. Special stains did not reveal any infectious organisms. Bacterial, fungal, and acid-fast tissue cultures were negative. Due to the rarity of leishmaniasis and leprosy in her geographic area, absence of travel history, and lack of specific features on pathology, additional specialty microbiologic testing was not performed. Blood tests for tuberculosis and coccidioidomycosis were negative. Standard laboratory evaluations showed stable leukopenia, and CSF analysis at the time of her skin biopsy did not show concern for malignancy. Workup to identify an associated autoimmune disease was negative. A diagnosis of RGD was rendered. Drug reaction was ruled out as the patient had been maintained on the same medical regimen which was not a well-described culprit medication, and the pathology did not show eosinophils. The family preferred conservative management with topical steroids and petrolatum. After several months, the lesions resolved with scarring and there was no evidence of recurrence at six months.

## 3. Conclusion

Our case demonstrates a dual diagnosis of RGD and ALL. Only a few reported cases detail RGD occurring in the pediatric age group (Table 1).

In 2015, Rosenbach and English proposed the umbrella term “reactive granulomatous dermatitis” to encompass several clinical and pathologic entities including PNGD, interstitial granulomatous dermatitis, and interstitial granulomatous drug reaction recognizing that granulomatous dermatitis is usually not a stand-alone diagnosis and rather a reaction pattern that occurs in the context of other systemic conditions [3]. There is a significant degree of clinical and histopathologic overlap among these entities.

Classically, RGD is described as skin-colored to erythematous umbilicated or crusted papules or nodules occurring in a symmetric distribution that favors the extremities. Multiple other morphologies including annular plaques, linear bands, and edematous or urticarial lesions have been described [3].

On histology, the findings of RGD may vary based on the age of the lesions sampled; features include a combination of neutrophilic infiltration, small vessel vasculitis, collagen degeneration, palisaded granulomas, and dermal fibrosis [3, 5]. The pathogenesis of RGD remains poorly understood. Several theories have been proposed including abnormal neutrophil activation, immune complex deposition, delayed-type hypersensitivity reaction, and low-grade vasculitis [2, 3].

RGD has been rarely reported in pediatric patients with inflammatory conditions including juvenile arthritis, systemic lupus erythematosus, and now, hematologic malignancy [6–9]. The correct identification of RGD is paramount, as it may provide insight into an undiagnosed underlying condition. The exclusion of infection is essential. Treatment is generally directed towards the underlying inflammatory condition, but agents such as topical, intralesional, and systemic steroids and colchicine and dapsone, among others have been reported [3, 10–13].

## Figures and Tables

**Table 1:** Among the cases listed, this case report is the only pediatric case reported in the context of hematologic malignancy.

Cases	Age and sex	Associated condition	Distribution	Clinical presentation
Henostroza-inga et al.	11 F	SLE	Limbs	Papular-erythematous and violaceous crusted vesicle
Biswas et al.	10 F	DM1, celiac	Foot, ankle	Tender papules
Hunt et al.	14 F	P-ANCA FSGN	Elbows, knees, buttocks, and hand	Papulonodules with crusting
Kwon et al.	7 F	Sarcoidosis	Back, elbows, knees, upper eyelids, hands, and feet	Nontender, firm, papules and plaques
Germanas et al.	12 F	SLE	Neck, arms, flank, abdomen, and thighs	Erythematous plaques
Nguyen et al.	15 F	JIA	Limbs	Painful, erythematous papules and nodules
Thakkar et al.	9 M	Acute myelocytic leukemia	Face, limbs	Erythematous, papular lesions
This case	3 F	Acute lymphoblastic leukemia	Limbs	Tender nodules and plaques with central ulceration

## Data Availability

No data were used to support this study.
